# *Caenorhabditis elegans* *daf-7* mutants exhibit burrowing behavior

**DOI:** 10.17912/micropub.biology.000172

**Published:** 2019-11-04

**Authors:** Araceli López-Puebla, Zyanya Mayoral-Peña, Kitzia Gómez-Cepeda, Fausto Arellano-Carbajal

**Affiliations:** 1 Facultad de Ciencias Naturales, Universidad Autónoma de Querétaro, México

**Figure 1:**
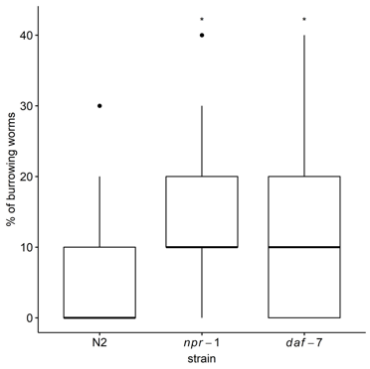
Percentage of burrowing worms on plates with eight perforations. Strains used were wild-type strain N2 (Bristol), DA609 [*npr-1(ad609*)], and CB1372 [*daf-7(e1372)]*. *P < 0.05 compared to N2 worms.

## Description

Burrowing was originally described for *C. elegans* as worms that tended to stay below the surface of the agar when maintaining high densities of nematodes on agar (Hodgkin and Doniach, 1997). The neuropeptide receptor NPR-1 regulates food-related behaviors (de Bono and Bargmann, 1998). The canonical TGF-β signaling pathway responds to environmental signals by regulating egg laying and feeding (Gumienny and Savage-Dunn, 2013). The TGF-β like ligand DAF-7 functions as a sensor of environmental conditions (Greer et al., 2008). As the aggregating mutant strain *npr-1* appeared to burrow more than the wild-type N2 strain (Hodgkin and Doniach, 1997; de Bono and Bargmann, 1998), it was suggested that aggregation preludes burrowing (Rogers et al., 2006). Considering that *daf-7* mutants also aggregate on food (Thomas et al., 1993; de Bono et al., 2002), we examined whether *daf-7* mutants also exhibit burrowing behavior.

The burrowing assay consisted in using 3.5-cm NGM plates with eight concentric perforations made at 0.875 cm from the edge of the plate. Perforations were made by puncturing the agar with a sterile 0.5 µl pipette tip until reaching the bottom surface of the dish. 10 nematodes were transferred to the plates after perforations had been made. The number of worms within the agar was counted after 2 h. Fifty experimental replicates for wild-type strain N2 and mutant strains *daf-7* and *npr-1* were done per assay. This number of replicates yielded a statistical power ≥ 0.85. All agar plates were prepared two days prior to the assays to ensure stability and reduce excessive moisture (Brenner, 1974). We discarded any plate that showed fractures in the agar’s surface. In order to compare burrowing between wild-type worms and each of the mutant strains we used Chi-square tests. We also calculated the odds ratio (OR) and 95% confidence intervals. Analysis were run in R 3.3.1 (R Core Team, 2015). Fisher exact p-values and odds ratios were calculated with R package Epitools.

Mutant strains *npr-1* and *daf-7* behaved similarly on the agar plates with perforations, where they were more likely to burrow than the wild-type strain (*npr-1* vs. N2: Chi2=4.79, p=0.03; OR=1.63, 95% CI=1.04-2.52; *daf-7* vs. N2: Chi2=5.23, p=0.02; OR=1.66, 95% CI=1.07-2.57) (Figure 1). Past studies have reported that the aggregating strain *npr-1* burrows more than the N2 strain (Hodgkin and Doniach, 1997; de Bono and Bargmann, 1998). We found that both aggregating strains *npr-1* and *daf-7* tended to exhibit similar burrowing behavior on agar plates when perforations were present. A study that focused on foraging proposed that burrowing was likely to occur as a way to avoid hyperoxia at the surface of the agar plate (Rogers et al., 2006). Considering that *daf-7* mutants avoid hyperoxia (Chang et al., 2006), the burrowing behavior observed in *daf-7* mutants might be further evidence that this behavior is induced in response to hyperoxia, as is known to explain aggregation behavior. We have tested only a single mutant allele for *daf-7*, therefore these data are preliminary. In addition, further testing of other genes acting in the TGF-β signaling pathway, for which there exist reports that their mutants exhibit aggregation behavior, such as *daf-1*, *daf-8* and *daf-14* (Thomas et al., 1993), would support our findings that TGF- β pathway regulates burrowing behavior.

## Reagents

The strains used in this study were wild-type strain N2 (Bristol), DA609 [*npr-1(ad609*)], and CB1372 [*daf-7(e1372)*]. All strains were provided by the Caenorhabditis Genetic Center. Worms were maintained at 18°C in NGM and were fed with *E. coli* OP50 (Brenner, 1974).
